# Shelf-life extension of soy sauce by using chitosan oligosaccharides combined with tea polyphenols

**DOI:** 10.1016/j.fochx.2023.100985

**Published:** 2023-11-07

**Authors:** Ying Zhu, Chao Gong, Saikun Pan, Shengjun Wu

**Affiliations:** a Jiangsu Key Laboratory of Marine Bioresources and Environment/Jiangsu Key Laboratory of Marine Biotechnology, Jiangsu Ocean University, Haizhou, China; b Co-Innovation Center of Jiangsu Marine Bio-industry Technology, Haizhou, China

**Keywords:** Chitosan oligosaccharides, Tea polyphenols, Shelf-life, Soy sauce

## Abstract

Chitosan oligosaccharides (COs) and tea polyphenols (TPs) have antioxidant and antibacterial activities. This study aims to explore the preservative effects of 0.1 % COs alone and combined with 0.08 % TPs on soy sauce during room-temperature storage. Soy sauce treated with 0.1 % COs alone and combined with 0.08 % TPs had lower total bacterial count, *Escherichia coli* count and pH, and higher amino acid nitrogen and overall likeness score than those of the control group during room-temperature storage. Treatment with 0.1 % COs combined with 0.08 % TPs extended the shelf life of soy sauce by at least 15 months compared with the control group. Results showed 0.1 % COs combined with 0.08 % TPs may be a feasible method to extend the shelf life of soy sauce during room-temperature storage.

## Highlights

• COs combined with TPs reduced bacterial growth in soys auce.
• COs combined with TPs retarded pH increase in soys auce.
• COs combined with TPs retarded AAN decrease in soys auce.
• COs combined with TPs maintained overall likeness of soys auce.

## 1 Introduction

Soy sauce is a traditional fermented seasoning that originated in China, and its unique flavour has been favoured by people. It has become a commonly used seasoning in Asian countries such as China, Japan and South Korea, and has gradually been accepted by Western countries (Devanthi & Gkatzionis, 2019). Soy sauce is mainly brewed from soybeans, wheat or bran, and salt through koji making, fermentation and other processes (Li et al., 2020). The ingredients of soy sauce are very complex. In addition to salt, various amino acids, sugars, organic acids, pigments and spices are present. Soy sauce is mainly salty but also has a fresh taste and aroma. It can increase and improve the taste of dishes but also add or change their colour (Diez, Eichelsheim, Mumm & Hall, 2020). The labouring people of ancient China mastered the brewing of soy sauce thousands of years ago. Soy sauce generally has two types, dark soy sauce and light soy sauce; light soy sauce is used for freshening, whereas dark soy sauce is used for enhancing colour (Soumaya, Imad, Shimar & Zul, 2021). Soy sauce is prone to deteriorate due to its high nutrition and moisture content. Therefore, a practical method must be developed for the preservation of soy sauce.

Chitosan oligosaccharide (CO) is an oligosaccharide product obtained by degrading chitosan through special biological enzyme technology, chemical degradation technology and microwave degradation technology, and has a polymerization degree between 2 and 20. COs are low-molecular-weight products with good water solubility. COs have many biological activities such as antioxidant (Kim et al., 2009), antibacterial and antitumor (Yu et al., 2022). Tea polyphenols (TPs) are a complex of multiple hydroxy phenolic compounds in tea, consisting of more than 30 phenolic substances. The main components of TPs are catechins and their derivatives, which are the main chemical components in tea with health functions. TPs have various physiological activities such as antioxidant (Wang, Liu, Wu, Zhang & Zhang, 2022), antibacterial (Li, Feng, Jin, Yang & Wang, 2022), hypolipidemic and hypoglycaemic (Tenore, Stiuso, Campiglia & Novellino, 2013).

Therefore, COs combined with TPs may have antibacterial and antioxidant activities, and inhibit soy sauce deterioration during room-temperature storage. In addition, data regarding the preservative effects of COs combined with TPs on soy sauce during room-temperature storage are limited. Thus, this paper aims to investigate the preservative effects of COs combined with TPs on soy sauce during room-temperature storage.

## 2 Methods and materials

### 2.1 Materials

Light soy sauce with 6.2 g/100 mL sodium content, 0.74 g/100 mL amino acid nitrogen (AAN) and initial pH 4.1 was purchased from Jiangsu Tongwan Brewing Co., Ltd. COs with a purity > 99 % were purchased from Xuanyu Biotechnology Co., Ltd., Suzhou, China. TPs with a purity > 99 % were purchased from Guangzhou Xinrurong Biotechnology Co., Ltd., Guangzhou, China. All other chemicals were of reagent grade.

### 2.2 Treatment of soy sauce

The test soy sauce samples were 108 plastic barrels of soy sauce, each barrel containing 1 L of soy sauce. The soy sauce samples were randomly assigned to three groups: Control, Treatment-1 (0.1 % COs) and Treatment-2 (0.1 % COs + 0.08 % TPs). The soy sauce samples were stored at room temperature (∼20 ℃) for 18 months.

### 2.3 Total viable count assay

The total bacterial count in the soy sauce was determined using a standard plate counting method (Yerlikaya, Gokoglu & Topuz, 2010). In a glass plate, 1 mL of soy sauce sample or diluted soy sauce sample was inoculated into a solid nutrient agar medium and incubated at 37 °C for 48 h. The bacterial colonies on the medium were counted and expressed as log colony forming unit (CFU)/mL.

### 2.4 Coliform group measurement

The soy sauce sample was made into a serial decimal dilution. One millilitre of the sample diluents with different dilutions was inoculated into Lauryl Sulfate Tryptose (LST) broth tubes. After incubation at 36 ℃ for 48 h, one loop of culture was taken from all the LST broth tubes that fermented and produced gas within 48 h using an inoculation ring, and then transferred to Brilliant Green Lactose Bile (BGLB) broth tubes. After inoculation, the BGLB broth test tube was incubated at 36 ℃ for 48 h, and the gas production was observed. The gas producer was the coliform positive tube according to the number of tubes confirmed positive for coliform bacteria at different dilutions. The most possible number (MPN) value of coliform bacteria per millilitre of soy sauce was reported according to the MPN table (Wang, Xie, Feng, Huang & Zhao, 2023).

### 2.5 pH measurement

The pH of the soy sauce were measured using a digital pH meter (PHS-3C, Shanghai Lichen Instrument Technology Co., Ltd., Shanghai, China) in accordance with the methods of Duan, Cherian and Zhao (2010).

### 2.6 Amino acid nitrogen assay

The soy sauce sample was titrated to pH 8.2 with 0.05 mol/L sodium hydroxide standard solution and then to pH 9.2 with 0.05 mol/L sodium hydroxide standard solution after adding formaldehyde. A blank test was also conducted. The result was expressed as g/100 mL (Wang et al., 2023).

### 2.7 Sensory evaluation

The soy sauce sample was sterilized at 90 ℃ for 10 min, cooled down and evaluated by 12 trained food research workers from Jiangsu Ocean University, China. The overall likeness score of the soy sauce sample was evaluated using the 1–9 descriptive hedonic scale: 9 means the highest sensory quality, 1 means the lowest sensory quality and 5 means the lowest acceptable score (Li, Wang, Fang & Li, 2013).

### 2.8 Statistical analysis

The soy sauce samples were analysed at 0, 1, 2, 3, 6, 12 and 18 months of room-temperature storage. All experiments were replicated thrice. All data were expressed by mean ± standard deviation. Origin 7.0 statistical analysis software was used for data collation and analysis. Paired sample *t*-test was used for significant difference analysis.

## 3 Results and discussion

### 3.1 Effect of COs and TPs on microbial growth

**Fig. 1:**
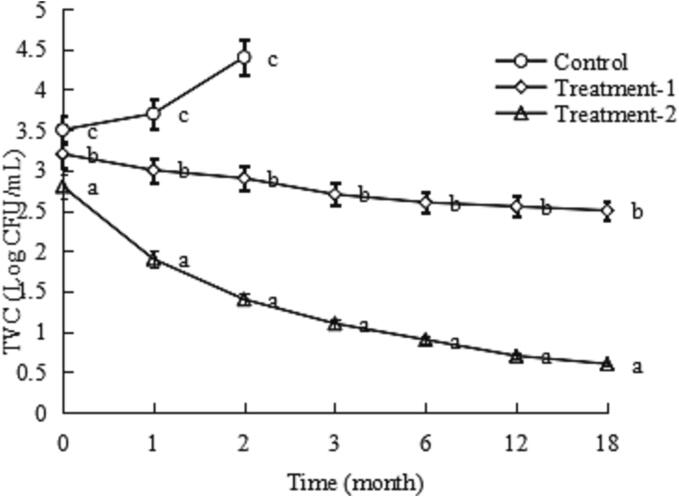
Effect of chitosan oligosaccharides alone (Treatment-1) or combined with tea polyphenols (Treatment-2) on the total viable count (TVC) of soy sauce during storage. Bars represent the standard deviation (n = 3).

**Fig. 2:**
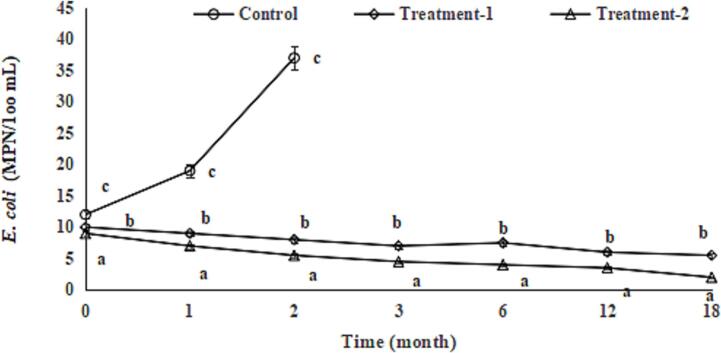
Effect of chitosan oligosaccharides alone (Treatment-1) or combined with tea polyphenols (Treatment-2) on *Escherichia coli (E. coli)* count of soy sauce during storage. Bars represent the standard deviation (n = 3).

The effects of COs and TPs on microbial growth in soy sauce during room-temperature storage are presented in Fig. 1, Fig. 2. The total viable count (TVC) and *Coliform* group of the soy sauce in the control group increased sharply, exceeded the GB upper limit, and lost edible and commercial values after 2 months of room-temperature storage, whereas the TVCs and *Coliform* groups of the soy sauce in Treatment-1 and Treatment-2 decreased during 18 months of room-temperature storage; this could be due to the antibacterial activities of COs (Yu et al., 2022) and TPs (Li et al., 2022). The differences in the TVCs and *Coliform* groups between Treatment-1 and Treatment-2 increased during 18 months of room-temperature storage; this could be due to the antibacterial activity of TPs (Li et al., 2022) and showed a synergistic effect of COs and TPs. Therefore, COs combined with TPs led to lower microbial growth in the soy sauce in treatment groups than in the control group (*P* < 0.05). Recently, COs have been used for the preservation of *Collichthys niveatus* (Zheng, He, Zhou & Xiong, 2019) and milk (Tsai, Wu & Su, 2000), whereas TPs have been used for the preservation of stewed beef chunks (Li et al., 2021, Yang et al., 2023), silver carp (Zhou, Liu, Liao, Wang & Xia, 2022), chilled cat spermatozoa (Wittayarat, Panyaboriban, Kupthammasan, Otoi & Chatdarong, 2022) and fresh beef (Bing et al., 2022). Moreover, Gao, Liu, Chen, Su and Mo (2016) fund that 0.06 g/100 mL COs combined with 0.3 g/100 mL TPs suppressed the growth of aerobic bacteria and *Coliform* group in chilled beef.

### 3.2 Effect of COs and TPs on pH

**Fig. 3:**
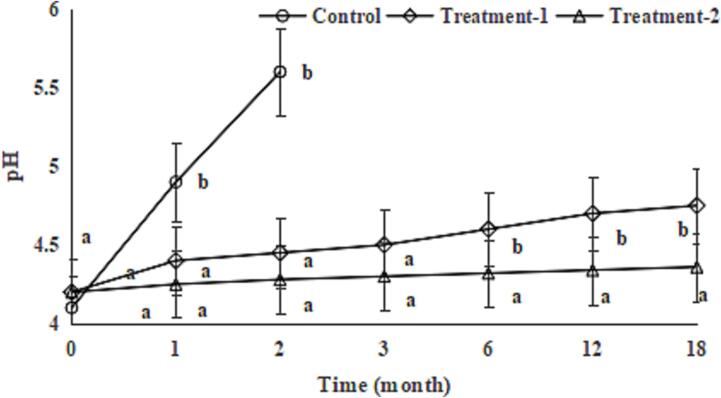
Effect of chitosan oligosaccharides alone (Treatment-1) or combined with tea polyphenols (Treatment-2) on pH of soy sauce during storage. Bars represent the standard deviation (n = 3).

pH is one of important indices of soy sauce, and the normal pH range for soy sauce is 3.8–5.4. During storage, due to the growth and metabolism of microorganisms, the amino acids in soy sauce undergo a decarboxylation reaction, generating primary ammonia and leading to an increase in pH. The pH of the soy sauce in the control group increased sharply and exceeded the upper limit (pH 5.4) and lost edible and commercial values after 2 months of room-temperature storage, the pH of the soy sauce in Treatment-1 increased slowly and the pH of the soy sauce in Treatment-2 remained unchanged during 18 months of room-temperature storage (Fig. 3); this also could be due to the antibacterial activities of COs (Yu et al., 2022) and TPs (Li et al., 2022). The differences in the pH values between Treatment-1 and Treatment-2 increased during 18 months of room-temperature storage; this could be due to the antibacterial activity of TPs (Li et al., 2022) and showed a synergistic effect of COs and TPs.

### 3.3 Effect of COs and TPs on amino acid nitrogen

**Fig. 4:**
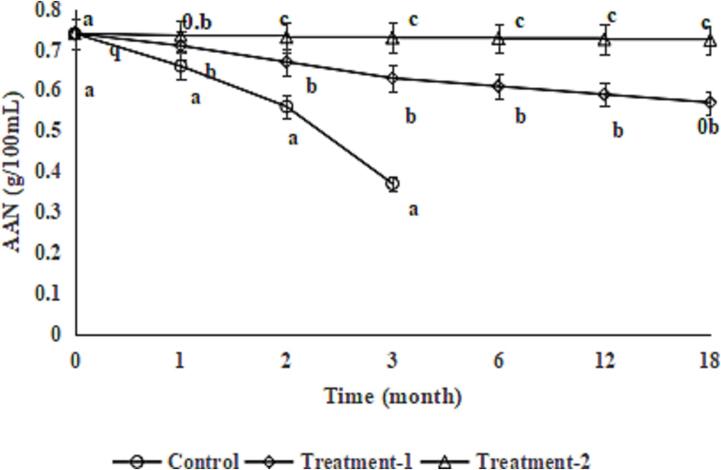
Effect of chitosan oligosaccharides alone (Treatment-1) or combined with tea polyphenols (Treatment-2) on amino acid nitrogen (AAN) of soy sauce during storage. Bars represent the standard deviation (n = 3).

AAN is an important indicator of soy sauce, and the lower limit of the national standard is 4.0 g/100 mL. During storage, due to the growth and metabolism of microorganisms, amino acids are consumed as nutrients for microorganisms, manifested by a decrease in AAN. The AAN of the soy sauce in the control group decreased sharply and exceeded the lower limit of the national standard and lost commercial value after 3 months of room-temperature storage, whereas the AAN of the soy sauce in Treatment-1and Treatment-2 decreased slowly during 18 months of room-temperature storage (Fig. 4); this also could be due to the antibacterial activities of COs (Yu et al., 2022) and TPs (Li et al., 2022). The differences in the AAN between Treatment-1 and Treatment-2 increased during 18 months of room-temperature storage; this also could be due to the antibacterial activity of TPs (Li et al., 2022) and showed a synergistic effect of COs and TPs.

### 3.4 Effect of COs and TPs on overall likeness score

**Fig. 5:**
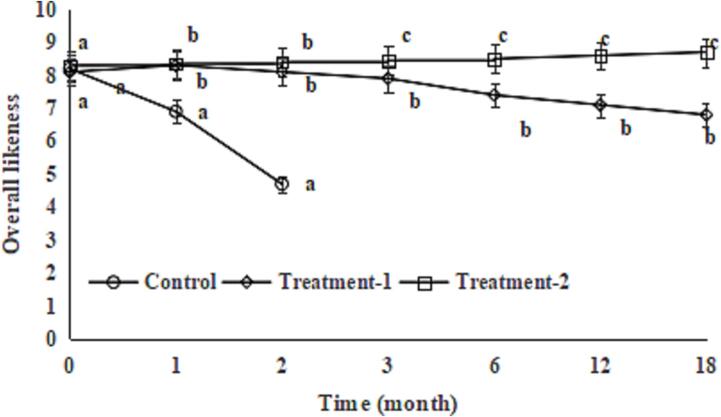
Effect of chitosan oligosaccharides alone (Treatment-1) or combined with tea polyphenols (Treatment-2) on overall likeness of soy sauce during storage. Bars represent the standard deviation (n = 3).

Sensory parameters are an important indicator of soy sauce. During storage, due to the endogenous enzyme and the growth and metabolism of microorganisms, colour, odour, taste and texture inevitably change, generally manifested as a decline in the sensory quality of soy sauce. The overall likeness score of the soy sauce in the control group decreased sharply, exceeded the minimum acceptable overall likeness score (5.0), and lost edible and commercial values after 3 months of room-temperature storage. The overall likeness score of the soy sauce in Treatment-1 decreased slowly, and the overall likeness score of the soy sauce in the Treatment-2 increased slightly during 18 months of room-temperature storage (Fig. 5); this also could be due to the antioxidant and antibacterial activities of COs (Kim et al., 2009, Yu et al., 2022) and TPs (Li et al., 2022, Wang et al., 2022). The differences in the overall likeness score between Treatment-1 and Treatment-2 increased during 18 months of room-temperature storage; this also could be due to the antioxidant and antibacterial activities of COs (Kim et al., 2009, Yu et al., 2022) and TPs (Li et al., 2022, Wang et al., 2022), and showed a synergistic effect of COs and TPs. Similarly, 0.06 g/100 mL COs combined with 0.3 g/100 mL TPs suppressed the decreases in L* and a* values of chilled beef (Gao et al., 2016).

## 4 Conclusions

COs combined with TPs effectively inhibited microbial growth, decreases in pH, AAN and overall likeness score of soy sauce during 18 months of room-temperature storage. The results indicated COs combined with TPs may be a practical method to inhibit the spoilage of soy sauce.

## CRediT authorship contribution statement

**Ying Zhu:** Methodology, Validation, Writing – review & editing. **Chao Gong:** Investigation, Software, Data curation, Writing – original draft. **Saikun Pan:** Resources, Supervision, Validation, Writing – review & editing. **Shengjun Wu:** Conceptualization, Validation, Writing – review & editing.

## Declaration of Competing Interest

The authors declare that they have no known competing financial interests or personal relationships that could have appeared to influence the work reported in this paper.

## Data Availability

The authors do not have permission to share data.
